# Rhodanine derivatives as potent anti-HIV and anti-HSV microbicides

**DOI:** 10.1371/journal.pone.0198478

**Published:** 2018-06-05

**Authors:** Cristina Tintori, Giulia Iovenitti, Elisa Rita Ceresola, Roberto Ferrarese, Claudio Zamperini, Annalaura Brai, Giulio Poli, Elena Dreassi, Valeria Cagno, David Lembo, Filippo Canducci, Maurizio Botta

**Affiliations:** 1 Department of Biotechnology, Chemistry and Pharmacy, University of Siena, Siena, Italy; 2 Laboratory of Microbiology and Virology, Ospedale San Raffaele, Milan, Italy; 3 Lead Discovery Siena S.r.l., Castelnuovo Berardenga, Siena, Italy; 4 Laboratory of Molecular Virology and Antiviral Research. Department of Clinical and Biological Sciences, University of Torino, Orbassano, Torino, Italy; 5 Department of Molecular Microbiology, University of Geneva, Geneva, Switzerland; 6 Sbarro Institute for Cancer Research and Molecular Medicine, Center for Biotechnology, College of Science and Technology, Temple University, Philadelphia, PA, United States of America; Istituto di Genetica Molecolare, ITALY

## Abstract

Although highly active antiretroviral therapies (HAART) remarkably increased life expectancy of HIV positive people, the rate of novel HIV-1 infections worldwide still represent a major concern. In this context, pre-exposure prophylaxis (PrEP) approaches such as vaginal microbicide gels topically releasing antiretroviral drugs, showed to have a striking impact in limiting HIV-1 spread. Nevertheless, the co-presence of other genital infections, particularly those due to HSV-1 or 2, constitute a serious drawback that strongly limits the efficacy of PrEP approaches. For this reason, combinations of different compounds with mixed antiviral and antiretroviral activity are thoroughly investigated Here we report the synthesis and the biological evaluation of a novel series of rhodanine derivatives, which showed to inhibit both HIV-1 and HSV-1/2 replication at nanomolar concentration, and were found to be active also on acyclovir resistant HSV-2 strains. The compounds showed a considerable reduction of activity in presence of serum due to a high binding to serum albumin, as determined through *in vitro* ADME evaluations. However, the most promising compound of the series maintained a considerable activity in gel formulation, with an EC_50_ comparable to that obtained for the reference drug tenofovir. Moreover, the series of compounds showed pharmacokinetic properties suitable for topical formulation, thus suggesting that the novel rhodanine derivatives could represent effective agents to be used as dual anti HIV/HSV microbicides in PrEP approaches.

## Introduction

Implementation of extensive patients’ treatment and prevention strategies in the global response against human immunodeficiency virus type 1 (HIV-1) epidemic has resulted in significant reduction of new infections worldwide. Nevertheless, sexually transmitted infections (STIs), including HIV-1, represent a major global burden, particularly in resource limited regions [1]. Moreover, since 2010, no significant reduction of novel HIV-1 infections was recoded in most countries, suggesting that current efforts, including the increase access to antiretroviral treatment are not sufficient to end the AIDS epidemic.

**Fig 1:**
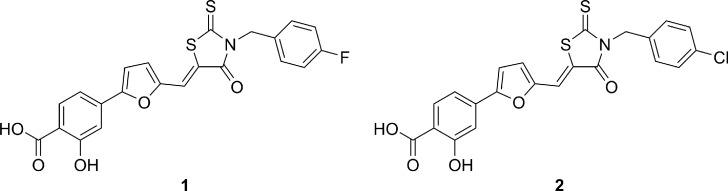
2D structures of HIV-1 inhibitors previously published.

Microbicides delivering antiviral compounds in the vagina, in the rectum or in the oral cavity, were shown to reduce HIV-1 transmission and can thus be considered effective tools to implement the global response to HIV-1. In fact, the CAPRISA 004 trial showed that 1% tenofovir (TFV) microbicide vaginal gel used as pre-exposure prophylaxis (PrEP) approach showed a 39% reduction of infection risk, demonstrating that topically used drugs can have a huge impact in limiting HIV-1 spread [2–7]. However, efficacy of currently planned PrEP approaches may be hampered by the presence of circulating strains already resistant to available antiretroviral agents (present in the PrEP preparations) or the co-presence of other genital infections that increase patient’s susceptibility to HIV-1 infection [8–11]. This is the case of genital infections caused by herpes simplex virus type 2 (and type-1 in fraction of genital infections). In some regions, especially in those with the highest prevalence of HIV-1 infection, such as in many African countries, up to 80% of young adults are serum-positive for genital herpes infection, with a significant fraction of herpes virus recurrences [12–15]. All currently available PrEP approaches, including vaginal gels containing antiretroviral or intravaginal rings with long-acting antiretroviral drugs or implantable and injectable formulations, selectively inhibit HIV-1 replication but have no or very limited activity against HSV-1 or 2 [16] thus their efficacy may be limited or not sufficient to prevent new infections especially in high prevalence settings for both viral infections. In our previous papers [17–19], we demonstrated that these molecules block very early step in HIV-1 replication, preventing viral entry into cells. This may be relevant to increase bioavailability of antiretroviral agents in topical preparations. In fact, almost all currently available PrEP approaches target intracellular steps of virus replication, suggesting that available drugs will have to penetrate both the mucosal barrier and the target cell membranes in order to be effective when used in topical preparations. During the screening of our *in house* library against a panel of sexually transmitted viruses, we surprisingly found that compounds 1 and 2 (Fig 1) [17–19], potently inhibited the replication of both HSV-1 and HSV-2, without any antiviral activity on HPV-16, a not enveloped virus particularly relevant in sexually transmitted infections. As a continuation of our previous work, a novel series of rhodanine derivatives was synthesized. Biological evaluation showed that these compounds are able to inhibit HIV-1 and HSV-1 and 2 replication at nanomolar concentration. We also characterized the binding affinity of these molecules to human albumin and their efficacy as antiviral agents in prototypical genital PrEP gel preparations. Furthermore, a preliminary ADME evaluation was performed by determining water solubility, passive membrane permeability and metabolic stability of selected derivatives.

## Results and discussion

### Chemistry

**Fig 2:**
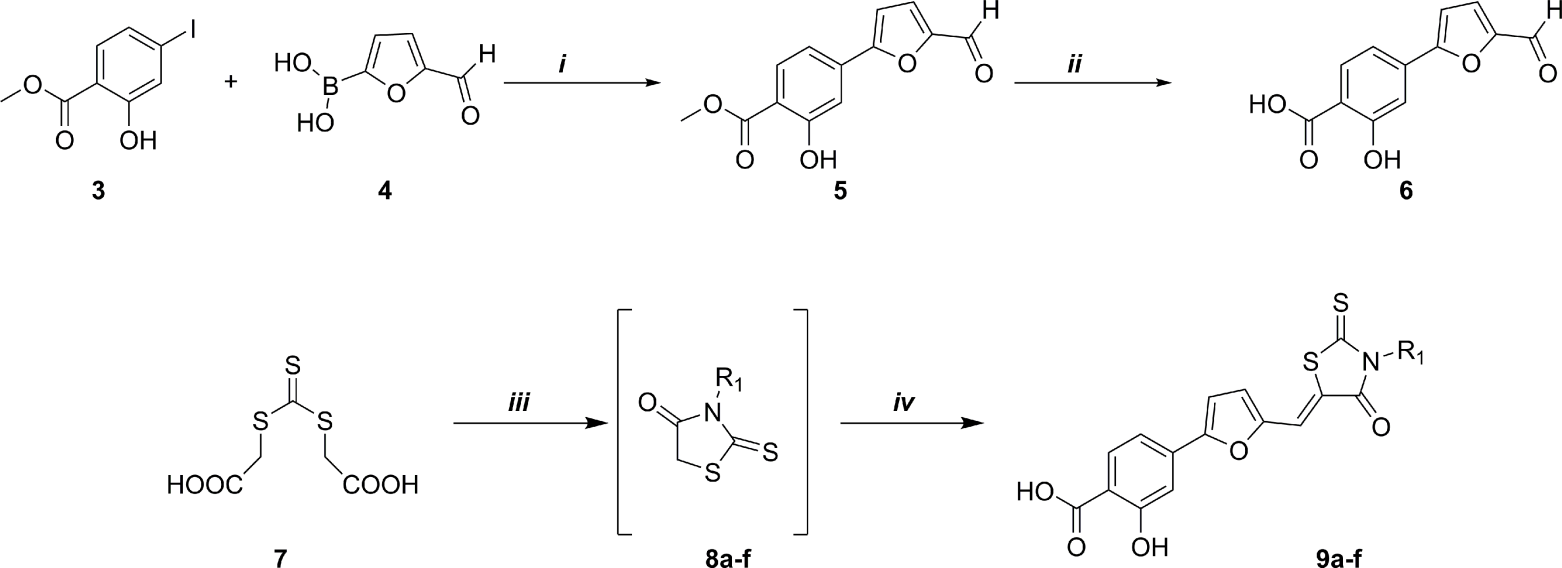
Synthesis of aldehyde 6 and derivatives 9a-f. Synthesis scheme of aldehyde **6**: *i)* Pd(PPh_3_)_2_Cl_2_, Na_2_CO_3_, DMF/EtOH, RT, 1h; ii) 1N NaOH (aq), MeOH/THF, reflux 2h. Synthesis scheme of derivatives **9a-f**: *iii*) DME, Et_3_N, MW (300 W), 90°C, 10 min. *iv*) aldehyde 6, MW (300 W), 110°C, 5 min.

As shown in Fig 2, the synthetic route to the final compounds **9a-f** first entailed the preparation of the target acid **6**. Methyl 4-(5-formylfuran-2-yl)-2-hydroxybenzoate (**5**) was obtained through Suzuki reaction between commercially available **3** (methyl 4-iodosalicylate) and compound **4** (5-formyl-2-furanylboronic acid), subsequent basic hydrolysis furnished the acid analogue (**6**). Derivatives **9a-f**, were synthesized using a recently published [18] multicomponent protocol that includes nucleophilic displacement between the opportune amine and trithiocarbonate (**7**) to form the substituted rhodanine intermediates (**8a-f**), followed by Knoevenagel condensation with aldehyde (**6**) catalysed by an excess of primary amine (Fig 2).

### Antiviral activity and cytotoxicity on TZM-bl cell line infected with HIV-1 laboratory strains

**Fig 3:**
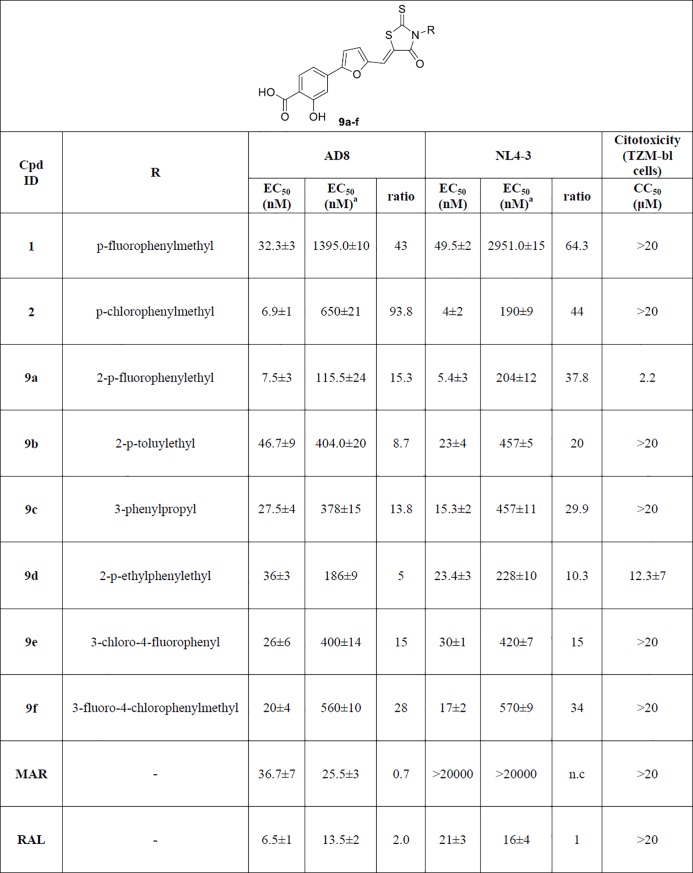
Antiviral activity of the novel series of rhodanine derivatives on TZM-bl cell line infected with two laboratory strains (NL4.3 and AD8). Maraviroc (MAR) and raltegavir (RAL) were used as reference compounds. Values represent mean±S.D of three independent experiments. Differences between pre-incubation in complete medium containing FBS are shown, together with the corresponding fold change (ratio). EC_50_ = Half maximal effective concentration. CC_50_ = Half maximal cytotoxic concentration. ^a^pre-incubation in complete medium containing fetal bovine serum (FBS).

The synthesized compounds (Fig 3) were tested *in vitro*, together with the parent compounds **1** and **2**, to evaluate their ability to inhibit HIV replication on human TZM-bl cells infected with HIV-1 NL4.3 (CXCR4-tropic strain) or AD8 (CCR5-tropic strain). All compounds showed antiviral activity at nanomolar concentrations with the parent compound **2** displaying the best inhibitory profile, having an EC_50_ of 6.9 nM and 4 nM on AD8 and NL4.3 HIV strains, respectively. Compound **9a** showed a comparable inhibitory effect (7.5 nM and 5.4 nM on AD8 and NL4.3 HIV strains, respectively) but a worst safety profile with respect to the other compounds. Indeed, almost all compounds of the series showed low cytotoxicity in TZM-bl cell line, with CC_50_ values higher than 20 μM. Only for compound **9a** and **9d** lower CC_50_ values were found (2.2 μM and 12.3 μM, respectively). Interestingly, all compounds displayed a reduction of antiviral activity when pre-incubation with virus was performed in presence of complete medium containing fetal bovine serum (FBS). Particularly, the activity of compound **2** against AD8 strain decreased 90 times in presence of serum, shifting from 6.9 nM to 650 nM. Also compound **1** showed a high loss of activity against NL4.3 strain, with an EC_50_ that moved from around 50 nM to 3 μM, corresponding to a 64-fold activity reduction. Compounds **9b-f** showed moderate to low loss of activity, in the range of 5 to 38 times. Commercially available drugs such as maraviroc [20] and raltegravir [21] did not show any alteration of antiviral activity when pre-incubated with serum.

### Antiviral activity and cytotoxicity on human CD4^+^ lymphocytes

The antiviral activity and cytotoxicity of the most promising compound (**2**) was evaluated also on freshly purified human CD4^+^ cells. CD4^+^ T lymphocytes were infected with the two reference laboratory wild type strains NL4.3 and AD8, as well as with two recombinant viruses bearing mutations of specific integrase residues (Q148H, G140S and Y143R) that confer resistance to raltegravir (RAL). As reported in Table 1, compound **2** was found to be active at low nanomolar concentrations (11–13 nM) on both NL4.3 and AD8 HIV strains also in CD4^+^ human lymphocytes, while showing a CC_50_ of 810 nM and thus a selectivity index of 62. Interestingly, in contrast to raltegravir, compound **2** maintained similar EC_50_ values also on integrase mutating recombinant viruses, thus suggesting that the strong antiviral activity of the series of rhodanine derivatives could not be ascribed to integrase inhibition [18].

**Table 1:** CC_50_ and EC_50_ values of the reference compound 2 and the control drug raltegravir (RAL) on human CD4^+^ T lymphocytes.

Compound	NL 4.3 WT EC_50_(nM)	AD8 WT EC_50_(nM)	Q148H, G140S EC_50_(nM)	Y143R EC_50_ (nM)	CC_50_ (nM)	SI
**2**	13±3	11±1	9±2	8±3	810±20	62
**RAL**	3±2	2±3	800±12	460±10	n.a	n.a

SI = Selectivity Index. EC_50_ = Half maximal effective concentration. CC_50_ = Half maximal cytotoxic concentration. Values represent mean±S.D of three independent experiments.

### Antiviral activity and cytotoxicity on Vero cell line infected with HSV-2 laboratory strain and HeLa infected with HPV-16 PsVs

The rhodanine derivatives were also tested *in vitro* to evaluate their antiviral activity toward HSV-2 on Vero cells and HPV-16 in HeLa cells. No antiviral activity was shown for all the compounds up to 50 μM for HPV-16. Instead, the compounds were subjected to a plaque reduction assay against HSV-2, in which they were pre-incubated with the virus for 1h at 37°C; then the mixtures were added on cells. As shown in Table 2, all the compounds were found to be active at nanomolar concentration and showed EC_50_ values lower than that obtained for acyclovir, which was used as reference compound. Particularly, the tested rhodanines proved to be up to 130-fold more active than acyclovir (compound **9b**) while presenting a very good safety profile, with selectivity indexes ranging from 600 (**9d**) to 9400 (**2**). Compound **2** showed the best HSV-2 antiviral profile, with an EC_50_ in the low nanomolar range (11.9 nM) and the highest selectivity index. Therefore, we further investigated the antiviral activity of this compound on HSV-1 and an acyclovir resistant strain of HSV-2.

**Table 2:** Antiviral activity of the rhodanine derivatives on Vero cell line infected with HSV-2.

Compound	HSV-2		Citotoxicity (Vero cells)	SI
	EC_50_ (nM)	EC_90_ (nM)	CC_50_ (μM)	
**1**	168±143	2107±918	224.2±78.5	1134
**2**	11.9±5.73	481±299	111.9±11.6	9403
**9a**	345±168	426±56.3	>300	>869
**9b**	4.89±1.59	57.8±22.6	10.8±.9.50	2209
**9c**	52.0±21.2	422±225	204.7±30.1	3936
**9d**	28.7±7.11	140±61.2	16.92±4.31	604.3
**9e**	50.3±10.5	206±85.5	>100	>1988
**9f**	26.4±11.1	153±159	7.87±2.98	298
**ACV**	622±21.1	-	732±46.2	1177

Acyclovir (ACV) was used as reference compound. Values represent mean±S.D of two independent experiments. EC_50_ = Half maximal effective concentration. EC_90_ = 90% maximal effective concentration. CC_50_ = Half maximal cytotoxic concentration. The selectivity index (SI) was calculated by dividing the CC_50_ by the EC_50_ value.

Compound **2** was found to be about 10 times less active on HSV-1 with respect to HSV-2 (Table 3). Anyway, this rhodanine derivative showed an EC_50_ (132 nM) slightly lower than that obtained for acyclovir (168 nM) and maintained a good safety profile (SI > 752.7). Moreover, compound **2** completely retained its antiviral activity toward the acyclovir resistant strain of HSV-2, suggesting a different mechanism of action and highlighting the potential of this series of rhodanines for the treatment of HSV-2 infections resistant to acyclovir. However, as expected, compound **2** showed a remarkable reduction of HSV-2 inhibitory activity when pre-incubated with the virus in presence of serum.

**Table 3:** Antiviral activity of compound 2 on Vero cell line infected with HSV-1, HSV-2 with serum and HSV-2 acyclovir resistant strains.

Compound	Virus	EC_50_ (nM)	Vero cell CC_50_ (μM)	SI
**2**	HSV-1	131±59.9	98±23.1	>752.7
**ACV**	HSV-1	168±41.3	698±72.5	4155
**2**	HSV-2 with serum	719±157.7	n.d.	n.d.
**2**	HSV-2 acyclovir resistant	10.7±6.34	111.9±11.6	>10,400
**ACV**	HSV-2 acyclovir resistant	31915±1159	732±46.2	22.93

Acyclovir (ACV) was used as reference compound. Values represent mean±S.D of two independent experiments. EC_50_ = Half maximal effective concentration. CC_50_ = Half maximal cytotoxic concentration. The selectivity index (SI) was calculated by dividing the CC_50_ by the EC_50_ value.

### Binding to bovine serum albumin in a cellular model

**Fig 4:**
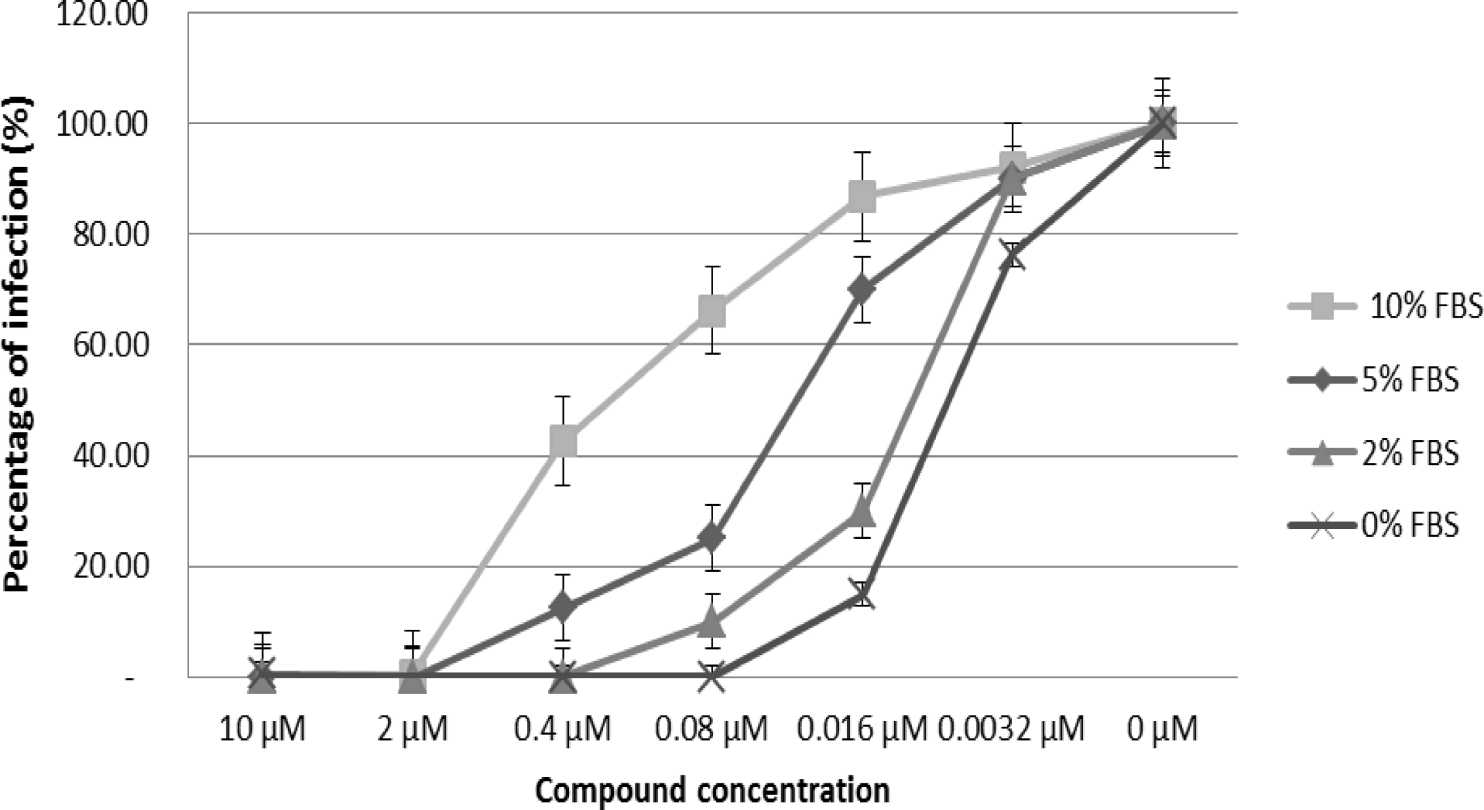
Effect of different concentrations of fetal bovine serum (0%, 2%, 5% and 10%) on the antiviral activity of compound 2. Compound **2** was tested in TZM-bl cells infected with AD8 HIV-1 laboratory strain.

To better elucidate the mechanism underlying the loss of activity observed for the rhodanine compounds during incubation with complete medium, the selected compound **2** and the control drug maraviroc were pre-incubated with the AD8 strain of HIV-1 in presence of concentrations of FBS ranging from 0% to 10%. As shown in Fig 4, the increase of FBS concentration from 2% to 10% corresponded to a progressive loss of activity, corroborating the inhibitory effect of serum on compound **2**. Such alteration was not seen on maraviroc, whose antiviral efficacy did not change with the modification of FBS concentration (data not shown).

**Fig 5:**
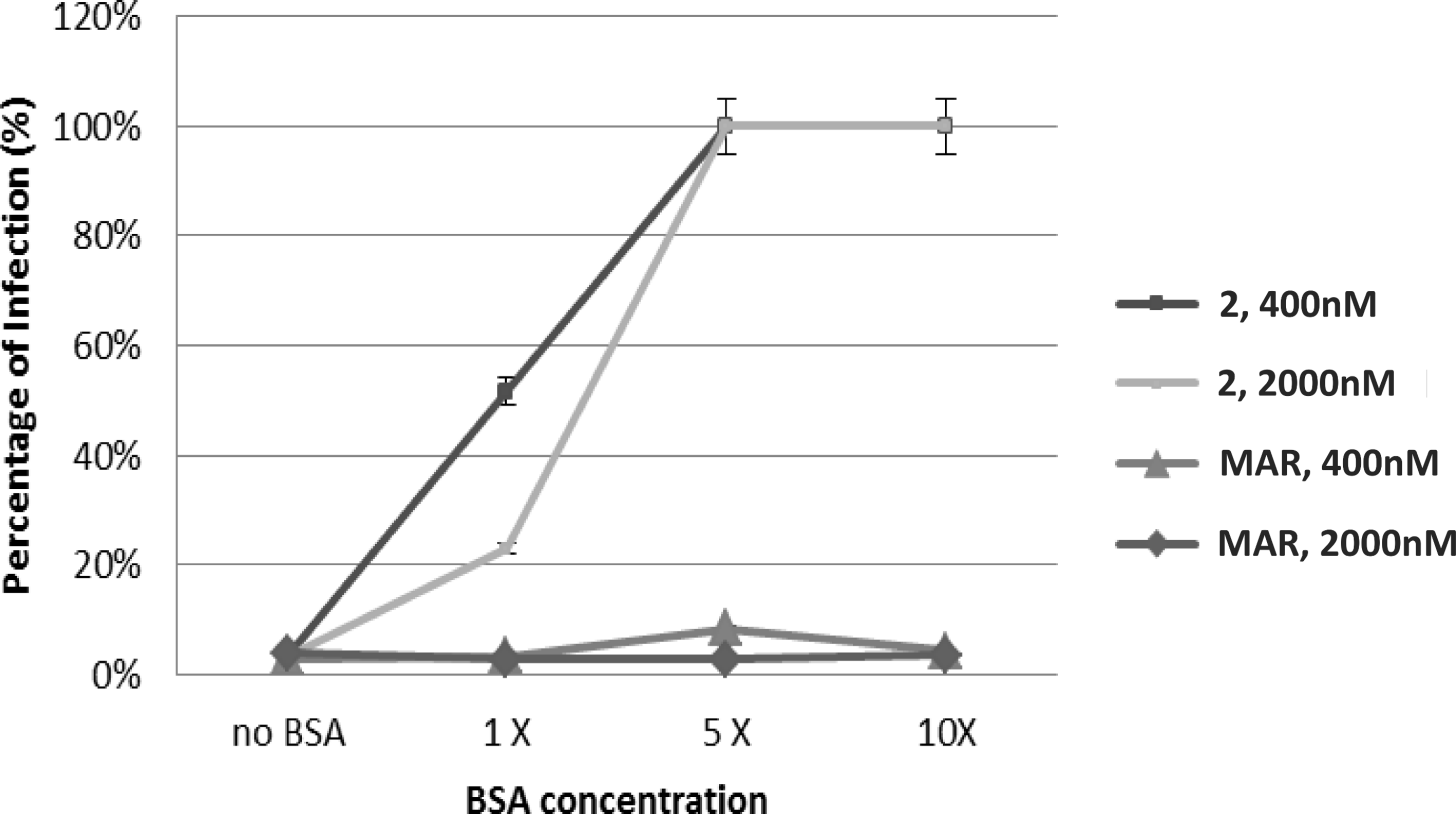
Effect of different concentrations of purified bovine serum albumin on the antiviral activity of compound 2 and maraviroc. Compounds were tested on TZM-bl cells infected with AD8 HIV-1 strain. No BSA = absence of BSA; 1X = 35 mg/mL BSA; 5X = 175 mg/mL BSA; 10X = 350 mg/mL BSA.

To evaluate if the inhibitory effect of serum on compound **2** was due to the binding to serum albumin, compound **2** and the reference drug maraviroc were pre-incubated with the virus and increasing concentrations of purified bovine serum albumin (BSA), starting from the physiological concentration present in the bovine serum up to ten-fold values. A physiological concentration of albumin altered only modestly the antiviral activity of compound **2** (20% loss), when tested at a concentration of 2 μM (Fig 5). However, at a concentration of 400 nM the reduction of compound’s activity was found to be stronger (about 50%) and increasing the BSA concentration of 5 to 10 folds led to a complete loss of activity. As expected, the antiviral activity of the control drug maraviroc was not influenced by any concentration of BSA.

### Antiviral activity of compound 2 in a microbicide gel formulation

**Fig 6:**
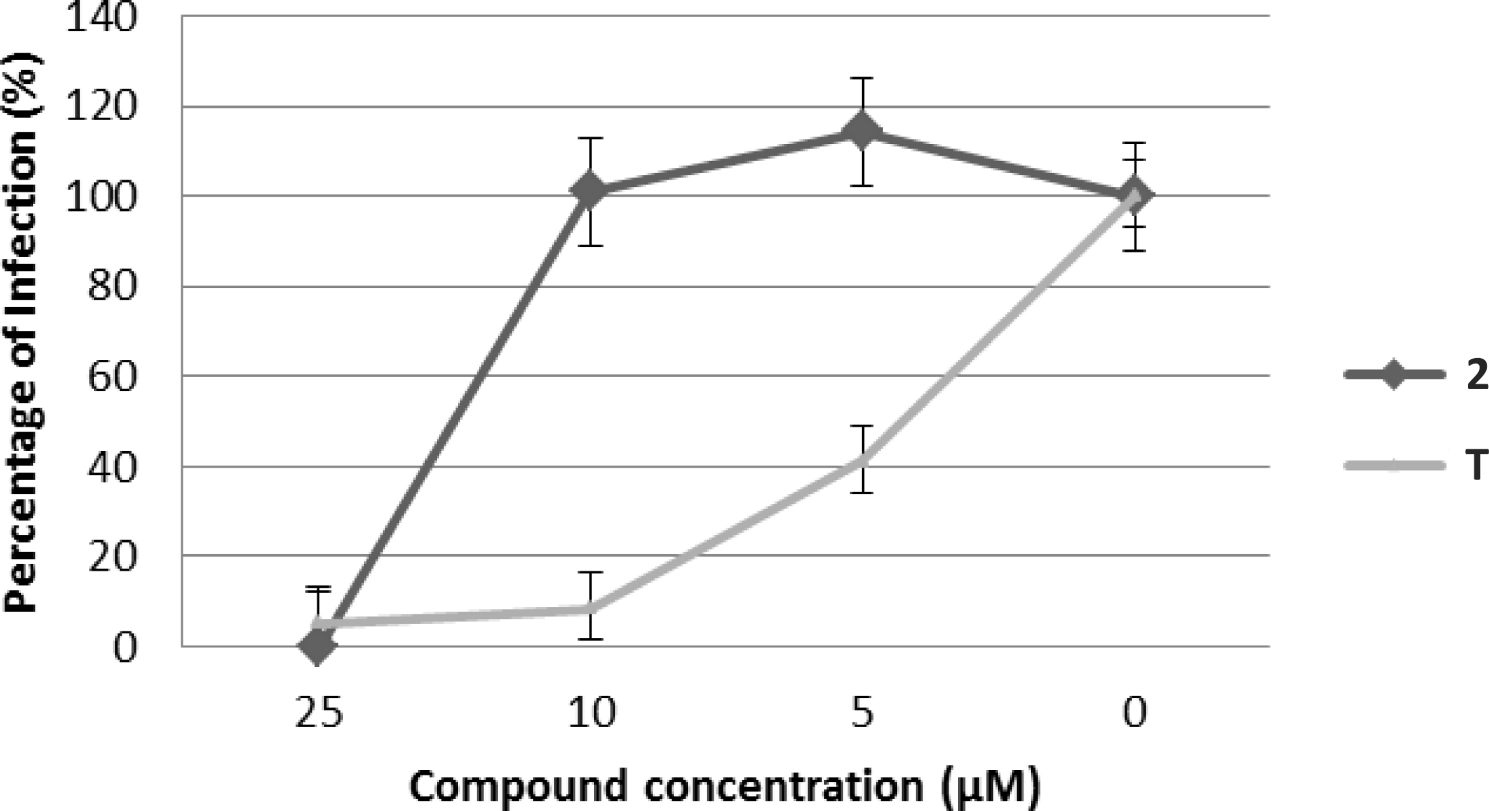
Anti-HIV-1 activity of compound 2 (black line) and tenofovir (T, grey line) in gel, formulation, in human TZM-bl cell line. Each concentration of both compounds was evaluated in triplicate.

Considered the promising antiviral activity of compound **2** on both HIV-1 and HSV-1/2, as well as the negative effect of serum on its efficacy, we envisioned that a topical administration of the compound would have overcome the problem of albumin binding and represented a suitable strategy for a pre-exposure prophylaxis (PrEP) approach for the treatment of HIV infection. For this reason we evaluated the activity of compound **2** and the reference compound tenofovir in a microbicide gel formulation using a transwell experiment. The usage of such experiment allowed to test the compounds in a gel formulation at a high concentration (50%) for all drug dilutions without affecting cell viability and antiretroviral activity evaluation. As shown in Fig 6, compound **2** maintained its antiviral activity in the gel formulation, despite the increase in EC_50_ observed in the transwell experiment (EC_50_ of 13 μM), which was however less than 3-fold higher than that observed for tenofovir (EC_50_ of 4.5 μM).

### In vitro ADME studies

#### Solubility, permeability and metabolic stability

Selected compounds from the rhodanine series were profiled *in vitro* for aqueous solubility (thermodynamics solubility), liver microsomal stability and membrane permeability (Table 4). Although the aqueous solubility of the compounds (ranging from 0.1 to 0.88 μg/mL) was found to be rather low, this aspect did not prevent the efficacy of the gel formulation according to the results obtain from solubility assessment (data not shown). In fact, the concentrations of the compounds in the pre-gel solutions obtained using our experimental conditions were identical to those of the control solution. In the same way, passive membrane permeability in a PAMPA assay indicated a low membrane permeability value for all compounds (ranging from 0.4 to 2.32 · 10^−6^ cm/s). However, this feature is considered to be drug-like, since it increases the time of exposure of the microbicide to the site of application, thus increasing its local activity. Moreover, stability tests disclosed that all compounds showed a good metabolic stability in human liver microsomes (>90%).

**Table 4:** Results of *in vitro* ADME analysis for selected rhodanine derivatives.

Compound	*In vitro* ADME				
	Water Solub. (μg/mL)	P*app* (1·10^-6^cm/sec)	Metabolic Stability (%)	K_d_ HSA (μM)	K_d_ BSA (μM)
**1**	0.88±0.10	1.28	>90%	0.63±0.1	0.68±0.1
**2**	0.52±0.13	1.26	>90%	0.96±0.1	0.63±0.1
**9a**	0.54±0.08	0.4	>90%	1.51±0.2	1.08±0.2
**9b**	0.10±0.08	2.32	>90%	1.57±0.2	1.55±0.3
**9c**	0.30±0.07	1.23	>90%	1.19±0.1	1.05±0.1
**9d**	0.25±0.09	1.50	>90%	1.09±0.9	0.99±0.9
**9e**	0.72±0.12	0.92	>90%	2.19±1.0	1.07±0.9
**9f**	0.73±0.14	0.87	>90%	2.23±1.2	1.09±0.9

Values represent mean±S.D of three independent experiments

#### Albumin binding fluorimetric assay

The selected method was first validated with drugs (paracetamol, diazepam and warfarin) having known K_d_ for human serum albumin (HSA) and experimental results were in good agreement with literature data [22] (S1 Table). Each compound analyzed by fluorimetric titration showed decreased intrinsic fluorescence of Tryptophan and the percentage of bound albumin (HAS and BSA) at different concentrations was calculated. The percentages obtained were plotted against the concentrations used and the obtained K_d_ values are reported in Table 4. Overall, all the compounds showed low K_d_ values, demonstrating a high binding affinity to serum albumin. In fact, it is well known that compounds showing K_d_ values lower than 10 μM are characterized by binding affinity to plasmatic proteins greater than 90% [22].

#### Storage Stability of compound 2 Gel Formulation

The sample, at final concentration of 50 μM, was found to be stable at 25°C stored in the dark. Compound **2** recovery after 1 month of storage amounted to about 100%. In addition, the apparent viscosity and pH of this gel remained stable during storage (283.31 ± 8.21 and 7.41 ± 0.08 respectively).

## Conclusions

Several studies confirmed the efficacy of topical PrEP approaches to limit the burden of novel HIV-1 infections and to enhance global efforts to eradicate this virus. However, several co-factors may limit the efficacy of PrEP approaches. Patients’ compliance due to the need of frequent or fastidious topical applications was initially shown as a critical cause of protection failure [9,10]. Therefore, the usage of long-acting formulations or slow releasing devices significantly ameliorated patients’ compliance and PrEP efficacy. Unfortunately, independently from the type of vehicle and release kinetic of current PrEP formulations, the presence of circulating strains already resistant to available antiretroviral agents (present in the PrEP preparations) or the co-presence of other genital infections such as those due to HSV-1 or 2, which increase patient’s susceptibility to HIV-1 infection, remain unsolved problems [11]. During HSV-1 or 2 infections, the mucosal barrier damage and the increased number of inflammatory cells susceptible to HIV-1 infection facilitate HIV-1 transmission. Interestingly, despite the very limited anti HSV-2 activity of tenofovir [16], in the CAPRISA 004 study, TFV PrEP reduced occurrence of HSV-2 infection by 50%, possibly enhancing the overall observed efficacy against HIV-1. Thus, multipurpose combinations of different compounds with antiviral and antiretroviral activity are intensively investigated, sometimes also associated with unintended pregnancy prevention compounds [23–28]. Compounds acting on both viruses such as the rhodanine derivatives described in this paper, represent effective agents to be used in candidate PrEP approaches without the need of dual-agents preparations. We demonstrated in our previous paper that these molecules block very early step in virus replication, preventing viral entry into cells. The inactivity against HPV-16, a not enveloped virus implicated in sexually transmitted infections suggests that the viral envelope could be implicated in the mode of action of our compounds. Preliminary data in our possess corroborate our hypothesis demonstrating that our compounds act on specific components of the viral envelope. These results will be reported in a subsequent more specific manuscript. Compounds targeting efficiently the entry of both HIV-1 or HSV-1 and 2 may represent a very attractive strategy to prevent target cell infection, with limited cell toxicity and reduced need of tissue penetration.

Moreover, PrEP devices undergoing clinical trials are based on already available antiretroviral molecules or their derivatives, thus running the risk of being ineffective to prevent transmission of previously selected drug-resistant strains from the donor patients. In fact, as expected, mutations which confer resistance to TFV have been reported in oral TFV PrEP regimens [11]. Similar observations may arise in the future in the case of other reverse transcriptase or integrase inhibitors PrEP-based devices, in parallel with the increased usage of molecules within these drug classes (with cross-resistance profiles) in currently used highly active antiretroviral therapies (HAART) worldwide, including developing countries. Thus, molecules with novel mechanisms of action and targeting very early steps of virus life cycle may represent potent anti-HIV-1 agents able to block the formation of a viral reservoir of chronically infected cells.

The activity of such inhibitors decreased of several times (from 20 to 100) once they were pre-incubated with serum before the *in vitro* assay. To better elucidate this behavior, the binding affinities of the new derivatives to the Human and Bovine Serum Albumin (HSA/BSA) were determined by a fluorescence-spectroscopy measurement and the K_d_ values were estimated. As a result, high affinity binding of the studied molecules to albumin was found, which could explain the loss of activity observed *in vitro* in the case of serum or purified recombinant albumin pre-incubation. Remarkably, the salicylic acid group resulted to be crucial for both antiviral activity and albumin binding [18]. These pharmacokinetic characteristics, which will be better explored in the future to modulate a long-acting release or activity of this compound even for systemic antiretroviral treatments, could be extremely useful to reduce toxicity of these novel compounds when used in topical preparations. Indeed, high locally active drug concentrations may be reached (favored also by the drug-like features observed in the ADME experiments) at the mucosal interface, with a significantly reduced risk of systemic toxicity due to the rapid albumin binding and inactivation of the compounds.

## Materials and methods

### Chemistry

#### General information

All commercially available chemicals were used as purchased. Anhydrous reactions were run under a positive pressure of dry N_2_. Thin-layer chromatography (TLC) was carried out using Merck TLC plates: silica gel 60 F254. Chromatographic purifications were performed on columns packed with Merck 60 silica gel, 23–400 mesh, for the flash technique. ^1^H and ^13^C NMR spectra were recorded at 400 MHz on a Bruker Avance DPX400 spectrometer. Melting points were measured using a Gallenkamp melting point apparatus and are uncorrected. Microwave irradiation experiments were conducted using a CEM Discover Synthesis Unit (CEM Corp., Matthews, NC, USA). The instrument consists of a continuous focused microwave power delivery system with operator-selectable power output from 0 to 300 W. The temperature of the contents of the vessel was monitored with a calibrated IR temperature control mounted under the reaction vessel. All experiments were performed using a stirring option, whereby the contents of the vessel are stirred by a rotating magnetic plate located below the floor of the microwave cavity and a teflon-coated magnetic stir bar in the vessel.

#### Methyl 4-(5-formylfuran-2-yl)-2-hydroxybenzoate (5)

Methyl-4-iodosalycilate **3** (1.00 mmol) and 5-formyl-2- furan boronic acid **4** were dissolved in 10 mL of DMF and 15 mL of EtOH. The reaction mixture was stirred for 10 minutes under N_2_, then Pd(PPh_3_)2 Cl_2_ (0.10 mmol) was added and finally Na_2_CO_3_ 2M (6.00 mmol). The reaction mixture (light-orange) was stirred under N_2_ at room temperature. After 1h the reaction went to completion (monitoring with TLC) and was quenched with H_2_O and 2N HCl, then EtOAc was added, and the mixture was stirred until the two layers became clear. The aqueous layer was extracted three times with EtOAc, then the organic phase were washed several times with H_2_O and brine, dried over Na_2_SO_4_, filtered and evaporated under reduced pressure. The crude product was purified by flash chromatography using PE/EtOAc = 4:1 as eluent to yield the wishes product **5** as a light orange solid (yield: 96%); mp = 150°C (decomposition); ^1^H NMR (CDCl3, 400 MHz): ð = 10.84 (s, 1H), 9.69 (s, 1H), 7.91–7.88 (d, 1H, *J =* 12 Hz), 7.40–7.39 (d, 1H, *J =* 4 Hz), 7.35–7.32 (m, 3H), 6.94–6.93 (d, 1H, *J =* 4Hz), 3.97 (s, 3H); ^13^C (CDCl_3_, 100 MHz): ð = 177.47, 161.69, 157.37, 152.53, 135.17, 130.58,122.56, 115.74, 113.73, 112.82, 109.84, 52.40, 29.59; MS (ES): m/z 245.0 [M-H]^-^; Anal. (C13H10O5) C, H, N.

#### 4-(5-Formylfuran-2-yl)-2-hydroxybenzoic acid (6)

Compound **5** was dissolved in 25 mL of CH_3_OH, then a solution of NaOH 1M (5.00 mmol) was added dropwise, after the reaction mixture was heated at reflux. The reaction mixture was stirred overnight until completion (TLC). Organic solvent was removed under reduced pressure, then some water was added, and the aqueous layer was extracted three times with Et_2_O; the aqueous layer was then acidified to pH 1 with HCl 6N and a precipitated appeared. (**6**) was obtained as a brown-red solid (yield: 95%); mp = 230°C (decomposition); ^1^H NMR (DMSO, 400 MHz): ð = 9.63 (s, 1H), 7.88–7.86 (d, 1H, *J =* 8 Hz), 7.66–7.65 (d, 1H, *J =* 4 Hz), 7.45–7.39 (m, 3H); ^13^C NMR (DMSO, 100 MHz): ð = 178.72, 171.68, 161.69, 156.82, 152.63, 135.11, 131.72, 125.14, 116.02, 114.06, 113.29, 111.53; MS (ES): m/z 231.0 [M-H].

#### General Procedure for the synthesis of final compounds 9a-f

To a solution of bis(carboxymethyl)trithiocarbonate (0.22 mmol) in DME (1.0 mL) were added TEA (0.22 mmol) and the opportune amine (0.22 mmol). The reaction mixture was heated at 90°C for 10 min under microwave irradiation. After this time, the aldehyde **6** (0.22 mmol) was added, and the mixture was heated at 110°C for 5 min under microwave irradiation. The reaction mixture was evaporated to dryness and the residue was additioned with MeOH and a drop of HCl 2N; the final rhodanine derivatives were obtained as a pure precipitate, isolated by filtration, washed with water and hexane, and finally dried under high vacuum.

#### (Z)-4-(5-((3-(4-fluorophenethyl)-4-oxo-2-thioxothiazolidin-5-ylidene)methyl)furan-2-yl)-2-hydroxybenzoic acid (9a)

(yield: 30%); Yellow solid. Mp = 292°C (decomposition), ^1^H NMR: (400 MHz, DMSO-*d*_6_) d = 7.92–7.90 (d, 1H, *J =* 8.0 Hz), 7.61 (s, 1H), 7.42–7.41 (d, 1H, *J =* 3.2 Hz), 7.38–7.33 (m, 3H),7.26–7.22 (m, 2H), 7.11–7.06 (m, 2H), 4.26.4.22 (m, 2H), 2.99–2.95 (m, 2H) ppm. ^13^C NMR (100 MHz, DMSO-*d*_6_): d = 194.20, 171.72,166.65, 161.84, 156.71, 154.49, 150.43, 134.83, 134.20, 131.78, 131.00,130.91, 123.19, 119.91, 118.46, 116.16, 115.72, 115.51, 113.76, 113.04,112.41, 45.63, 31.68 ppm. MS (ES): m/z 468.0 [M-H]^-^. HPLC: tr = 4.58 min; conditions: temp = 25°C, mobile phase composed of (A)70% acetonitrile and (B) 30% water with 0.5% formic acid at a flowrate of 1.0 mL/min; purity: 96.5%.

#### (Z)-2-hydroxy-4-(5-((3-(4-methylphenethyl)-4-oxo-2-thioxothiazolidin-5-ylidene)methyl)furan-2-yl)benzoic acid (9b)

(yield: 79%); Orange solid. Mp = 258°C (decomposition);1H NMR: (400 MHz DMSO-*d*_6_) d = 7.91–7.89 (d, 1H, *J =* 8.0 Hz), 7.60 (s, 1H), 7.42–7.33 (m, 4H), 7.10–7.08 (m, 4H), 4.21–4.18 (m, 2H), 2.92–2.88 (m, 2H), 2.25 (s, 3H) ppm. 13C NMR (100 MHz, DMSO-*d*_6_): d = 194.03, 178.56, 171.56, 166.68, 161.84, 156.79, 150.52, 135.99, 134.93, 131.77, 129.44, 128.88, 122.95, 120.15, 118.36, 115.56, 114.37, 113.86, 112.91, 112.52, 11.32, 45.70, 32.16, 20.96 ppm. MS (ES): m/z 464.0 [M-H]^-^. HPLC: tr = 4.65 min; conditions: temp = 25°C, mobile phase composed of (A) 70% acetonitrile and (B) 30% water with 0.5% formic acid at a flow rate of 1.0 mL/min; purity:95.9%.

#### (Z)-2-hydroxy-4-(5-((4-oxo-3-(3-phenylpropyl)-2-thioxothiazolidin-5-ylidene)methyl)furan-2-yl)benzoic acid (9c)

(yield: 91%); Orange solid. Mp = 248°C (decomposition); 1HNMR: (400 MHz, DMSO-*d*_6_) d = 7.90–7.88 (d, 1H, *J =* 8.4 Hz), 7.61 (s,1H), 7.44–7.43 (d, 1H, *J =* 4.0 Hz), 7.36–7.32 (m, 2H), 7.28–7.14 (m, 5H), 4.04–4.01 (m, 2H), 2.66–2.62 (m, 2H), 1.98–1.92 (m, 2H) ppm. 13C NMR (100 MHz, DMSO-*d*_6_): d = 194.23, 171.74, 166.91, 161.79, 156.60, 150.53, 141.20, 134.94, 131.77, 128.68, 128.55, 126.30, 122.99, 120.28, 118.28, 115.53, 113.49, 113.06, 112.39, 44.39, 32.72, 28.16 ppm. MS (ES): m/z 464.0 [M-1]^-^. HPLC: tr = 4.36 min; conditions: temp = 25°C, mobile phase composed of (A) 70% acetonitrile and (B) 30% water with 0,5% formic acid at a flow rate of 1.0 mL/min; purity: 96.8%.

#### (Z)-4-(5-((3-(4-ethylphenethyl)-4-oxo-2-thioxothiazolidin-5-ylidene)methyl)furan-2-yl)-2-hydroxybenzoic acid (9d)

(yield: 80%); Brown solid. Mp = 273°C (decomposition);^1^H NMR: (400 MHz, DMSO-*d*_6_) d = 7.89–7.87 (d, 1H, *J =* 8.0 Hz), 7.58 (s, 1H), 7.42–7.41 (d, 1H, *J =* 3.6 Hz), 7.34–7.30 (m, 2H), 7.12–7.11 (m, 5H), 4.18–4.14 (m, 2H), 2.90–2.86 (m, 2H), 2.57–2.52 (q, 2H, *J =* 7.6 Hz), 1.15–1.11 (t, 3H, *J =* 7.6 Hz) ppm. ^13^C NMR (DMSO-*d*_6_100 MHz): d = 193.44, 171.74, 166.58, 161.80, 156.66, 150.42, 149.51, 142.40, 135.13, 134.85, 131.71, 128.92, 128.28, 123.10, 119.97, 118.35,115.49, 113.54, 113.01, 112.39, 45.69, 32.16, 28.16, 15.98 ppm. MS (ES): m/z 478.0 [M-H]^-^. HPLC: tr = 4.23 min; conditions: temp = 25°C, mobile phase composed of (A) 70% acetonitrile and (B) 30% water with 0.5% formic acid at a flow rate of 1.0 mL/min; purity:96.5%.

#### (Z)-4-(5-((3-(3-chloro-4-fluorophenyl)-4-oxo-2-thioxothiazolidin-5-ylidene)methyl)furan-2-yl)-2-hydroxybenzoic acid (9e)

(yield 30%) red solid, ^1^H NMR (400 MHz, DMSO-*d*_6_) δ11.32 (s, 1H), 7.89 (d, *J =* 8.1 Hz, 1H), 7.77 (d, *J =* 6.4 Hz, 1H), 7.68 (s, 1H), 7.62 (t, *J =* 8.9 Hz, 1H), 7.54–7.47 (m, 1H), 7.43 (d, *J =* 3.3 Hz, 1H), 7.36–7.32 (m,3H)ppm; ^13^C NMR (DMSO-*d*_6_100 MHz): δ 198.0, 171.60, 167.62, 156.59, 155.80, 152.63, 152.07, 135.06, 134.53, 129.32, 126.76, 125.48, 124.06, 122.43, 120.60, 117.57, 115.92, 115.62, 111.62, 111.29, 103.95 ppm MS (ES): m/z 474.0 [M-H]^-^. HPLC: tr = 4.61 min; conditions: temp = 25°C, mobile phase composed of (A) 70% acetonitrile and (B) 30% water with 0,5% formic acid at a flow rate of 1.0 mL/min; purity:97.1%.

#### (Z)-4-(5-((3-(4-chloro-3-fluorobenzyl)-4-oxo-2-thioxothiazolidin-5-ylidene)methyl)furan-2-yl)-2-hydroxybenzoic acid (9f)

(yield 57%) brown solid, ^1^H NMR (400 MHz, DMSO-*d*_6_) δ 11.28 (s, 1H), 7.85 (d, *J =* 8.0 Hz, 1H), 7.62 (s, 1H), 7.52 (t, *J =* 7.8 Hz, 1H), 7.39 (s, 1H), 7.36–7.24 (m, 3H), 7.15 (d, *J =* 8.0 Hz, 1H), 5.18 (s, 2H)ppm, ^13^C NMR (101 MHz, DMSO-*d*_6_) δ 204.26, 181.73, 176.87, 171.78, 168.65, 166.82, 165.98, 160.40, 146.86, 144.67, 141.68, 140.82, 135.19, 133.36, 129.92, 129.20, 128.86, 126.69, 126.19, 125.22, 123.56, 123.03, 122.07, 56.51ppm. MS (ES): m/z 488.0 [M-H]^-^. HPLC: tr = 4.28 min; conditions: temp = 25°C, mobile phase composed of (A) 70% acetonitrile and (B) 30% water with 0.5% formic acid at a flow rate of 1.0 mL/min; purity:96.6%.

### Antiviral activity and cell toxicity

#### Phenotypic analyses with fully replicating recombinant HIV-1 strains and clonal viral variants selected in patients failing integrase inhibitors on TZM-bl cells and human CD4+ lymphocyte

The human TZM-bl indicator cell line (obtained from the NIH AIDS reagents program, cat. Nr 8129 (www.aidsreagent.org) and maintained at 37°C and 5% CO_2_ in Dulbecco’s modified Eagle’s medium (DMEM) medium containing 10% fetal bovine serum, 50 μg/mL penicillin, and 50 μg/mL streptomycin. The HIV-1 laboratory strains NL(AD8) (NIH AIDS reagents program, cat.nr 11346), NL4.3 (NIH AIDS reagents program, cat. Nr. 114) were titrated as previously reported. Briefly, serial 5-fold dilutions of each virus were made in quadruplicate wells in 96-well culture plates, in a total volume of 100 μL of growth medium (DMEM), for a total of 8 dilution steps. Freshly trypsinized cells (20,000 cells in 100 μL of growth medium containing 75 μg/mL DEAE-dextran) were added to each well, and the plates were incubated at 37°C in a humidified 5% CO_2_-95% air environment. After 48 h of incubation, the medium was removed and viral infection was quantified using a β-galactosidase (CPRG) assay (Roche). Twenty thousand TZM-bl cells/well were seeded in 96-well plates in complete DMEM supplemented with 30 μg/mL DEAE-dextran (Sigma-Aldrich). Three hundred times the 50% tissue culture infective dose (TCID50)/mL of each strain was pretreated for 1 h at 37°C with six serial dilutions (20000 nM to 3.2 nM) of each compound and then added to the cells, as previously described [29–32]. Vehicle (0.1% dimethyl sulfoxide [DMSO])-treated cells served as a negative control. A CCR5 inhibitor (maraviroc) and an integrase inhibitor (raltegravir) were used as positive-control drugs. After 2 days, viral infection was quantified using a CPRG assay (Roche). The inhibitory curves were fitted by nonlinear regression, allowing for the calculation of the 50% inhibitory concentration (IC_50_) using the Prism software. To evaluate the cell toxicity of the compounds, the metabolic XTT [2,3-bis-(2-methoxy-4-nitro-5-sulfophenyl)-2*H*-tetrazolium-5-carboxanilide] test (Sigma-Aldrich) was performed according to the manufacturer’s instructions.

The antiviral activity and cytotoxicity of compounds **2**, **9e** and **9f** was tested also on freshly purified human CD4^+^ T lymphocytes obtained from healthy blood donors (informed consent available) as described previously on HIV laboratory strain NL4.3 and AD8 [33,34].

#### HSV-1 and HSV-2 antiviral activity in vero cells

African green monkey fibroblastoid kidney cells (Vero, ATCC CCL-81) were grown as monolayers in Eagle's minimal essential medium (MEM) (Gibco/BRL, Gaithersburg, MD) supplemented with 10% heat inactivated fetal calf serum (FCS) and 1% antibiotic-antimycotic solution (Zell Shield, Minerva Biolabs GmbH, Berlin, Germany).

Clinical isolates of HSV-1 and HSV-2 were kindly provided by Prof. M. Pistello, University of Pisa, Italy. HSV-1 and HSV-2 strains were propagated and titrated by plaque assay on Vero cells. A HSV-2 strain with phenotypic resistance to acyclovir was generated by serial passage in the presence of increasing concentrations of acyclovir, as previously described [35].

Acyclovir was purchased from Sigma Aldrich (Milan, Italy).

The effect of the rhodanine derivatives on HSV infection was evaluated by plaque reduction assay. Vero cells were pre-plated 24 h in advance in 24-well plates at a density of 105 cells. Increasing concentrations of compounds were mixed with HSV-2 (MOI 0.001 pfu/cell) or HSV-2 acyclovir resistant (MOI 0.001) or HSV-1 (MOI 0.0005) or and incubated for 1 hour at 37°C. The mixtures were subsequently added to the cells, which were then incubated at 37°C for 2 h. The virus inoculum was then removed and the cells washed and overlaid with a medium containing 1.2% methylcellulose (Sigma). After further incubation at 37°C for 24 h (HSV-2 and HSV-2 R Acy) or 48 h (HSV-1), cells were fixed and stained with 0.1% crystal violet in 20% ethanol and viral plaques counted. The effective concentration producing 50% reduction (EC_50_) and 90% reduction (EC_90_) in plaque formation was determined using Prism software by comparing drug-treated with wells treated with medium and solvent. The selectivity index (SI) was calculated by dividing the CC_50_ by the EC_50_ value.

All results are presented as the mean values from three independent experiments. The EC_50_ values for inhibition curves were calculated by regression analysis using the software GraphPad Prism (GraphPad Software, San Diego, California, U.S.A.) by fitting a variable slope-sigmoidal dose–response curve. Acyclovir was purchased from Sigma Aldrich (Milan, Italy).

#### Vero cell viability

Cell viability was measured using the MTS [3-(4,5-dimethylthiazol-2-yl)-5-(3-carboxymethoxyphenyl)-2-(4-sulfophenyl)-2H-tetrazolium] assay. Cell cultures were seeded in 96-well plates were incubated with different concentrations of compounds in triplicate under the same experimental conditions described for the antiviral assays. Cell viability was determined using the CellTiter 96 Proliferation Assay Kit (Promega, Madison, WI, USA) according to the manufacturer's instructions. Absorbances were measured using a Microplate Reader (Model 680, BIORAD) at 490 nm. The effect on cell viability at different concentrations of the compound was expressed as a percentage, by comparing absorbances of treated cells with those of cells incubated with culture medium and equal volumes of vehicle. The 50% cytotoxic concentrations (CC_50_) and 95% confidence intervals (CIs) were determined using Prism software (Graph-Pad Software, San Diego, CA).

### Human papilloma pseudovirus (HPV16 PsV) production

Plasmids and 293TT cells used for pseudovirus (PsV) production were kindly provided by John Schiller (National Cancer Institute, Bethesda, MD). 293TT cell line, derived from human embryonic kidney cells transformed with the simian virus 40 (SV40) large T antigen, was cultured in DMEM (Gibco-BRL, Gaithersburg, MD) supplemented with heat inactivated 10% FCS (Gibco- BRL), Glutamax-I 1% (Invitrogen, Carlsbad, CA) and nonessential amino acids 1% (Sigma Aldrich, Steinheim, Germany). HPV-16 PsVs were produced as described in Cagno et al 2015. 293TT cells were transfected with plasmid expressing the papillomavirus major and minor capsid proteins (L1 and L2, respectively), together with a reporter plasmid expressing GFP. Capsids were allowed to mature overnight in cell lysate; the clarified supernatant was then loaded on top of a density gradient of 27 to 33 to 39% Optiprep at room temperature for 3 h. The material was centrifuged at 28000 rpm for 16 h at 4°C in an SW41.1 rotor (Beckman Coulter, Inc., Fullerton, CA) and then collected by bottom puncture of the tubes. Fractions were inspected for purity in 10% sodium dodecyl sulfate (SDS)–Tris–glycine gels, titrated on 293TT cells to test for infectivity by GFP detection, and then pooled and frozen at -80°C until needed. The L1 protein content of PsV stocks was determined by comparison with bovine serum albumin standards in Coomassie-stained SDS-polyacrylamide gels.

### HPV-16 inhibition assay in HeLa cells

HeLa, Human Adenocarcinoma cells purchased from ATCC CCL-2, were cultured with DMEM (Gibco-BRL, Gaithersburg, MD) supplemented with heat inactivated 10% FCS (Gibco- BRL), Glutamax-I 1% (Invitrogen, Carlsbad, CA). Compounds were serially diluted and incubated with HPV-16 (MOI 0.1) for 1 h at 37°C. Then the mixture was added to 8E4 HeLa cells grown as monolayers in a 96-well plate. Three days post-infection, cells were observed with a confocal microscope Zeiss LSM 510 and positive cells were counted.

#### Inhibition of antiviral activity by fetal bovine serum

To evaluate the binding of compounds to plasma proteins also in a cell based assay, the infection with the HIV laboratory strain AD8 was performed in absence or in presence of different concentrations of Fetal Bovine Serum (FBS): 2%, 5% and 10%. The representative compound **2** was pre-incubated at different concentrations ranging from 10 μM to 3.2 nM with 300 TCID50 /mL of AD8 in DMEM supplemented with 50 μg/mL penicillin, 50 μg/mL streptomycin, 30 μg/mL DEAE-dextran and FBS at the concentration described above. Maraviroc was used as control. After an hour the mixture was added to 40.000 TZM-bl cells/well plated in a 96-well plate and after two days the infection was quantified using a CPRG assay (Roche) as previously described.

#### Inhibition of antiviral activity by purified bovine serum albumin

To confirm the binding of compound **2** to serum albumin, infection was performed in presence of different concentrations of purified bovine serum albumin (BSA, Sigma Aldrich) starting from the concentration that is usually present in FBS serum (35 mg/mL, 1X) and increasing to a five-fold concentration (175 mg/mL) up to a ten-fold concentration (350 mg/mL). In this experiment the pre-incubation between HIV and two selected concentrations of compound **2** and maraviroc (2000 nM and 400 nM) was performed in DMEM without FBS and supplemented with BSA at the concentrations described above. After one hour, the mixture was added to 40.000 TZM-bl cells/well plated in DMEM supplemented with 50 μg/mL penicillin, 50 μg/mL streptomycin, 30 μg/mL DEAE-dextran and 10% FBS in a 96-well plate. After two days the infection was evaluated through a CPRG assay.

### ADME assay

#### Chemicals and excipients

All solvents, L-α-phosphatidylcholine, hydroxyethylcellulose (HEC), and propionic acid were from reagents, were from Sigma-Aldrich Srl (Milan,Italy). Dodecane was purchased from Fluka (Milan, Italy). Pooled Male Donors 20 mg/mL HLM were from BD Gentest-Biosciences (San Jose, California). Milli-Q quality water (Millipore, Milford, MA, USA) was used. Hydrophobic filter plates (MultiScreen-IP, Clear Plates, 0.45 μm diameter pore size), 96-well microplates, and 96-well UV-transparent microplates were obtained from Millipore (Bedford, MA, USA).

#### UV/HPLC-MS method

LC analyses were performed by Agilent 1100 LC/MSD VL system (G1946C) (Agilent Technologies, Palo Alto, CA) constituted by a vacuum solvent degassing unit, a binary high-pressure gradient pump, an 1100 series UV detector and a 1100 MSD model VL benchtop mass spectrometer was used. The Agilent 1100 series mass spectra detection (MSD) single-quadrupole instrument was equipped with the orthogonal spray API-ES (Agilent Technologies, Palo Alto, CA). Nitrogen was used as nebulizing and drying gas. The pressure of the nebulizing gas, the flow of the drying gas, the capillary voltage, the fragmentor voltage and the vaporization temperature were set at 40 psi, 9 L/min, 3000 V, 70 V and 350°C, respectively. UV detection was monitored at 254 nm. The LC-ESI-MS determination was performed by operating the MSD in the negative ion mode. Spectra were acquired over the scan range m/z 50–1500 using a step size of 0.1 u. Chromatographic analysis was performed using a Kinetex EVO C18 100A column (150 x 4.6 mm, 5 μm particle size) at room temperature. Analysis was carried out using a gradient elution of acetonitrile (ACN) and an aqueous solution (HCOOH 0.1%v/v): t = 0min ACN 5%, t = 3min ACN 5%, t = 12min ACN 95%, t = 25 min ACN 95%. The analysis was performed at flow rate of 0.6 mL/min and injection volume was 20 μL.

#### Aqueous solubility

Each solid compound (1 mg) was added to 1 mL of water. Each sample was mixed at 20°C, in a shaker water bath for 24 h. The resulting suspension was filtered through a 0.45 μm nylon filter (Acrodisc). The concentration of solubilized compounds weres determined by UV/LC-MS (performed in triplicate) by comparison with the appropriate calibration curve that was obtained from samples of the compound dissolved in methanol at different concentrations.

#### Parallel Artificial Membrane Permeability Assay (PAMPA)

Donor solution (0.5 mM) was prepared by diluting 1 mM dimethylsulfoxide (DMSO) compound stock solution using phosphate buffer (pH 7.4, 0.025 M). Filters were coated with 5 μL of a 1% (w/v) dodecane solution of L-α-phosphatidylcholine. Donor solution (150 μL) was added to each well of the filter plate. To each well of the acceptor plate were added 300 μL of solution (50% DMSO in phosphate buffer). All compounds were tested in three different plates on different days. The sandwich was incubated for 5 h at room temperature under gentle shaking. After the incubation time, the plates were separated, and samples were taken from both receiver and donor sides and analyzed using UV/HPLC-MS gradient method above reported. Permeability (Papp) for PAMPA, were calculated according to the following equation, obtained from Wohnsland and Faller [36] and Sugano [37] equation with some modification in order to obtain permeability values in cm s-1,

$$P_{app}=\frac{V_{D}V_{A}}{\left(V_{D}+V_{A}\right)At}-\ln\left(1-r\right)$$

where V_A_ is the volume in the acceptor well, V_D_ is the volume in the donor well (cm^3^), A is the “effective area” of the membrane (cm^2^), t is the incubation time (s) and r the ratio between drug concentration in the acceptor and equilibrium concentration of the drug in the total volume (V_D_+V_A_). Drug concentration is estimated by using the peak area integration.

#### Metabolic stability in HLM (Human Liver Microsomes)

The incubation mixture (total volume of 500 μL) was constituted by the following components: HLM (0.2 mg/mL), a NADPH regenerating system (NADPH 0.2 mM, NADPH^+^ 1 mM, D-glucose-6-phosphate 4 mM, 4 unit/mL glucose-6-phosphate dehydrogenase and MgCl_2_ 48 mM), 50 μM of each compound in DMSO and phosphate buffer (pH 7.4, 25 mM, up to a final volume of 500 μL). The mixture was incubated at 37°C for 1 h. The reaction was cooled down and quenched with acetonitrile (1.0 mL). After centrifugation (4000 rpm, 10 min), the supernatant was taken, dried under nitrogen flow, suspended in 100 μL of methanol and analyzed by UV/LC-MS to determine the percentage of compound that was not metabolized.

#### Solubility assessment

The solubilizing capacity of formulation media was measured in the presence of 2% v/v of DMSO. Stock solution of compound **2** and tenofovir, were added to obtain a final concentration of 50, 25 and 10 μM in the final volume of 1 mL of buffer solution (pH 7.4 25 mM). After sonication, the samples were shacked in a shaker bath at room temperature for 24 h to reach equilibrium conditions. Each pre-gel solution was analyzed before and after filtration by a 0.45-μm nylon filter (Acrodisc). The solubilized compound was determined using LC-UV-MS method above reported.

#### Gel formulation

All the semi-solid formulations were prepared adding at the pre-gel solution reported above hydroxyethyl cellulose (HEC) (1.8% w/v). In all samples, 0.2% v/v propionic acid was added to aqueous solution in order to preserve form bacteria and mold contamination. HEC was dispersed in the pre-gel solution at 25°C under magnetic stirring, overnight.

#### Storage stability and rheological characterization

Gel containing compound **2** was stored at 25°C in the dark. At predetermined data points, the formulations were visually inspected to evidence aggregate formations and the pH was measured. Rheological evaluations were performed using a rotational viscometer (Bohlin Model Visco 88, Bohlin Instruments, UK). Appropriate measuring spindles (C14 DIN) were used during viscosity measurements. Samples were loaded into the cup and allowed to equilibrate for 10 min at desired temperature (25 ± 0.5°C) and apparent viscosity (mPa s) was determined using a shear rate of 552 s^-1^ over a 1 month period.

#### Binding fluorimetric assay

The binding of each compounds to HSA (human serum albumin) and BSA (bovine serum albumin) were monitored by fluorescence spectroscopy in order to determine the dissociation constant (Kd). A quantitative analysis of the potential interaction was performed by fluorimetric analysis using 96 multiwell plates: in each well a fixed concentration of HSA or BSA (10μM in phosphate buffer 1 mM), was added with different amounts of tested compound (0.1μM to 500μM by stock solutions in DMSO). Plates were gently shacked and after allowing 30 minutes at room temperature for equilibration, after excitation at 295nm, spectra were recorded from 300 to 400 nm with a Perkin Elmer EnVision Multilabel Reader 2014 spectrofluorimeter, spectra were acquired with EnVision Manager ver.1.13 software. The obtained fluorescence quenching percentages were plotted against drug concentrations and the relative K_d_ values were obtained using GraphPad software (version 6.0).

### Antiviral activity of compound 2 in a gel formulation

Antiviral activity of compound **2** on NL4.3 HIV strain was evaluated also in a gel formulation. To reproduce more physiological conditions, TZM-bl cells were seeded into transwells and the gel was applied onto the cell monolayer at 50% fixed concentration with serial drug dilutions as previously described. Briefly, 4x10^4^ TZM-bl cells were plated into each transwell apical chamber of a 24-well plate (pore size 3μm and diameter 6,5 mm, Corning) containing medium in the bottom plate and cultured overnight. For efficacy testing, 60 TCID50 of virus was added to each apical well in the presence of 100 μL of compound **2** gel, tenofovir gel or blank gel at the final drug concentrations of 25μM, 10 μM and 5 μM. Inhibition of infection was determined as previously based on deviations from blank gel.

## Supporting information

S1 Table Binding studies realized to validate the used fluorimetric assay. (PDF)
