# Supplementary material for: Rhodanine derivatives as potent anti-HIV and anti-HSV microbicides
Source: PLoS One. 2018 Jun 5;13(6):e0198478. doi: 10.1371/journal.pone.0198478 (PMC5988308; doi:10.1371/journal.pone.0198478)
Supplement: S1 Table — (PDF) [file pone.0198478.s001.pdf]

**S1 Table. Binding studies realized to validate the used fluorimetric assay.**

| Drug | Exp. data | Literature data | Reference |
|---|---|---|---|
| | HSA | | |
| | Kd (μM) | Kd (μM) | |
| Paracetamol | 564.7 ± 352.3 | 1279.0 ± 690.0 | [1] |
| Diazepam | 43.29 ± 5.1 | 28.0 ± 4.0 | [1] |
| Warfarin | 8.40 ± 0.7 | 6.8 ± 1.5 | [1] |

## References


1. Parikh HH, McElwain K, Balasubramanian V, Leung W, Wong D, Morris ME, et al. A rapid spectrofluorimetric technique for determining drug-serum protein binding suitable for high-throughput screening. Pharm Res. 2000; 17: 632-637.
